# Reframing spinal cord injury rehabilitation in fragile contexts: a multi-level systems framework for Lebanon

**DOI:** 10.1080/16549716.2026.2711533

**Published:** 2026-08-17

**Authors:** Nour El Hoda Saleh, Ibrahim Naim, Salem Hannoun, Linda Abou-Abbas, Dalia Khachman, Samar Rachidi

**Affiliations:** a Doctoral School of Sciences and Technology, Lebanese University, Hadat, Lebanon; b Clinical and Epidemiological Research Laboratory, Faculty of Pharmacy, Lebanese University, Hadat, Lebanon; c Department of Research, Health, Rehabilitation, Integration and Research Center (HRIR), Beirut, Lebanon; d Medical Imaging Sciences Program, Division of Health Professions, Faculty of Health Sciences, American University of Beirut, Beirut, Lebanon; e Faculty of Medical Sciences, Lebanese University, Beirut, Lebanon

**Keywords:** Universal Health Coverage, disability inclusion, health systems strengthening, low- and middle-income countries, crisis-affected settings

## Abstract

**Background:** Spinal cord injury (SCI) requires long-term, multidisciplinary rehabilitation to optimize functioning and participation. In fragile health systems, however, rehabilitation services are often fragmented, under-resourced, and inconsistently accessible. This Current Debate paper examines SCI rehabilitation in Lebanon as an example of how health system fragility constrains rehabilitation delivery and disability inclusion.

**Methods:** Using an International Classification of Functioning, Disability and Health (ICF)-informed approach, we developed a multi-level conceptual framework through structured narrative synthesis of 35 studies, contextual analysis of 10 policy documents, and iterative expert interpretation by a multidisciplinary team. The framework integrates evidence from rehabilitation systems literature with contextual analysis of the Lebanese healthcare system; its structure and prioritization reflect the authors’ interpretive synthesis and professional experience rather than a fully systematic or validated methodology.

**Results:** The proposed framework is organized across three interconnected levels: macro-level governance and financing, meso-level health system organization and workforce capacity, and micro-level functioning, self-management, and community participation. Health system fragility is conceptualized as a cross-cutting determinant influencing rehabilitation access, continuity of care, implementation capacity, and long-term outcomes. We argue that rehabilitation in crisis-affected settings cannot rely solely on incremental service expansion. Instead, rehabilitation should be reframed as a systems-level, disability-inclusive intervention addressing structural and contextual determinants of functioning.

**Conclusion:** The framework provides a context-sensitive starting point for strengthening rehabilitation systems in comparable low- and middle-income settings; its broader scalability and generalizability beyond Lebanon remain conceptual at this stage and require empirical and stakeholder validation.

## Paper Context

**Main findings:** Health system fragility acts as a cross-cutting determinant that shapes rehabilitation access, continuity of care, and long-term functioning for people with spinal cord injury in Lebanon.

**Added knowledge:** This paper introduces a multi-level framework, informed by international evidence and expert interpretation, that integrates governance, health system organization, and community participation while explicitly incorporating fragility as a cross-cutting determinant of rehabilitation outcomes.

**Global health impact for policy and action:** The proposed framework offers a scalable starting point for strengthening disability-inclusive rehabilitation policies and resilient rehabilitation systems in fragile and crisis-affected low- and middle-income countries.

## Key messages

- SCI rehabilitation in Lebanon is fragmented, inequitable, and highly sensitive to system fragility.
- Current global rehabilitation models insufficiently address structural constraints in crisis-affected settings.
- Rehabilitation must be reframed as a multi-level system intervention, not a standalone clinical service.
- An ICF-informed framework, derived from evidence synthesis and expert interpretation, can guide integrated action across governance, service delivery, and community levels; its scalability beyond Lebanon requires empirical validation.

## Background

Spinal cord injury (SCI) is a life-altering condition associated with long-term disability and profound impacts on functioning, autonomy, and quality of life [1], representing a substantial and growing global disability burden [2]. While advances in acute medical care have significantly improved survival rates, long-term outcomes remain highly dependent on access to comprehensive, multi-disciplinary rehabilitation, and constrained neurosurgical and trauma-management capacity in many low- and middle-income countries (LMICs) continues to shape the population subsequently requiring long-term rehabilitation [3–6].

Rehabilitation is increasingly recognized as an essential health service and a pillar of Universal Health Coverage (UHC), yet systems in many LMICs remain fragmented and under-resourced [7]. Lebanon exemplifies these challenges: an extremely privatized, fragmented health system with a historical lack of rehabilitation integration into primary care [8,9], compounded by economic collapse, political instability, and workforce ‘brain drain’ [10,11] – barriers to rehabilitation workforce capacity documented across other LMICs as well [12,13].

In Lebanon, individuals with SCI face unaffordable specialized care, an inaccessible physical environment despite existing legislation, and persistent stigma [14,15], echoing constraints reported among displaced and refugee populations in the region [16]. Addressing these interactions requires moving beyond a purely medical model of disability; the International Classification of Functioning, Disability and Health (ICF) frames disability as arising from the interaction between a health condition and the environment [17,18].

Existing initiatives such as WHO Rehabilitation 2030 emphasize service and workforce expansion, and community-based rehabilitation promotes inclusion, but both largely assume stable systems and are rarely integrated. This leaves a critical gap: current models insufficiently address the structural constraints of fragility – weak governance, economic collapse, fragmented delivery – leaving rehabilitation episodic and clinically focused. In response, this paper proposes an ICF-informed, multi-level framework for SCI rehabilitation in fragile health systems, arguing for a shift from service-expansion toward integrated strategies addressing structural determinants of disability, particularly relevant to LMICs facing protracted crises.

## The status quo: fragmentation and system failure

SCI rehabilitation in Lebanon is characterized by discontinuity, with services concentrated in acute hospitals and urban centers, leaving rural populations underserved [8,9,15,19] – a pattern echoed in Tunisia, Tanzania, Mongolia, and other LMICs, where infrastructure and workforce shortages similarly limit access [20–23]. Historically, interventions have focused on isolated inputs – such as assistive devices or short-term therapy – without addressing systemic barriers [9], resulting in:

- Preventable complications, including pressure injuries and infections from lack of continuity of care [24,25].
- Loss of functional gains, as environmental barriers limit post-discharge participation [26].
- Financial hardship from out-of-pocket payments and NGO-dependent care [11,27], mirrored in other fragile, donor-dependent rehabilitation settings [28,29].

The absence of a national SCI registry further limits evidence-based planning – a gap also reported in Tanzania, Nepal, and other lower-middle-income settings, where limited outcome data constrains rehabilitation planning [30–32].

## Methods

This Current Debate paper used an integrative approach combining literature synthesis, contextual policy analysis, and expert interpretation to identify factors shaping SCI rehabilitation in fragile health systems and synthesize them into a framework relevant to Lebanon. Given the exploratory, interpretive nature of this contribution, the approach was not intended as a systematic review or a formally validated framework-development methodology.

A structured PubMed search (January–March 2026) identified 35 studies addressing SCI rehabilitation, rehabilitation systems, and disability-inclusive care in LMICs and fragile settings; all 35 are cited throughout in support of the corresponding framework domains (search strategy and study list: Supplementary Material S1–S2). Additional studies were identified through reference screening and recent reviews on rehabilitation workforce and financing barriers [12,13,28,29,33].

A parallel review of ten national and international policy and contextual documents (Supplementary Material S3) informed the governance, financing, and service-organization domains.

Findings were interpreted using the ICF and refined through iterative discussion among a multidisciplinary author team (physiotherapy, rehabilitation, public health, disability research, and health systems; more than a decade of collective professional experience), converging on three interconnected levels – macro-level governance and financing, meso-level health system organization and service delivery, and micro-level functioning and participation – with health system fragility as a cross-cutting determinant across all domains.

Importantly, while the literature synthesis and policy analysis provided the evidentiary basis, the framework’s final structure and prioritization reflect the interpretive judgment and professional experience of the author team rather than a fully systematic or empirically validated synthesis; it should be understood as an expert-informed conceptual model, grounded in but not mechanically derived from the reviewed evidence – consistent with the interpretive scope of a Current Debate contribution. Supplementary Table S4 summarizes the evidence-to-theme mapping supporting framework development.

## The proposed multi-level action framework

**Figure 1.**
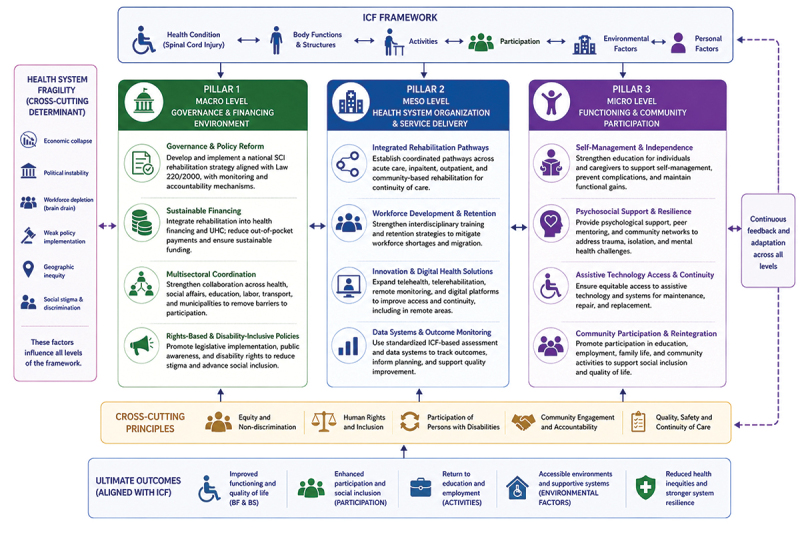
Proposed ICF-Informed multi-level action framework for spinal cord injury rehabilitation in fragile health systems.

Existing frameworks emphasize service expansion and workforce development but often assume stable health systems; in fragile settings such as Lebanon, rehabilitation is additionally constrained by economic instability, workforce shortages, and limited financing. We propose an ICF-informed framework treating health system fragility as a cross-cutting determinant of rehabilitation outcomes, organized across three dynamically linked levels: (1) macro-level governance and financing, (2) meso-level health system organization and service delivery, and (3) micro-level functioning and community participation (Figure 1).

## Pillar 1: macro-level governance and financing environment

The macro-level addresses governance, financing, and policy conditions shaping accessibility, equity, and sustainability. Key actions:

- Governance and policy reform: Operationalize a national SCI rehabilitation strategy aligned with Law 220/2000, with implementation mechanisms and accountability structures.
- Sustainable financing mechanisms: Reduce reliance on fragmented funding streams and high out-of-pocket costs by integrating rehabilitation within broader health financing and UHC initiatives, consistent with global recommendations for financing innovation in LMICs [28,34].
- Multisectoral coordination: Strengthen collaboration across health, social affairs, education, labor, and municipal sectors, reflecting evidence that health-system-level facilitators shape rehabilitation access across countries [29].
- Rights-based, disability-inclusive policies: Promote legislative implementation and disability-rights approaches supporting participation and reducing stigma.

## Pillar 2: meso-level health system organization and service delivery

The meso-level addresses organization, coordination, and delivery of services. Key actions:

- Integrated rehabilitation pathways: Link acute, inpatient, outpatient, and community-based care to support continuity, addressing service-organization barriers documented across LMICs and consistent with functional gains from multidisciplinary outpatient programs for chronic SCI [23,35–37].
- Workforce development and retention: Strengthen interdisciplinary training and retention strategies to mitigate the impact of workforce migration [12,13,38].
- Innovation and digital health: Expand teleconsultation, telerehabilitation, and remote monitoring to improve access and continuity, particularly in underserved areas, consistent with growing LMIC evidence [39–41].
- Data systems and outcome monitoring: Promote standardized ICF-based documentation for outcome tracking and quality improvement [42].

## Pillar 3: micro-level functioning and community participation

The micro-level addresses functioning, self-management, and community participation, explicitly foregrounding SCI-specific secondary complications and long-term surveillance rather than treating them as background considerations. Key actions:

- Self-management and secondary complication prevention: Strengthen patient/caregiver education and structured, lifelong follow-up for SCI-specific complications – neurogenic bladder and bowel dysfunction, pressure injuries, spasticity, and autonomic dysfunction – supported by participatory education approaches shown to improve occupational engagement [25,43–46].
- Psychosocial support, resilience, and sexual health: Integrate psychological support, peer mentoring, and sexual health counselling addressing trauma, isolation, and mental health challenges [47].
- Assistive technology access and continuity: Ensure equitable access to appropriate assistive technologies alongside systems for maintenance, repair, replacement, and long-term follow-up.
- Community participation and reintegration: Promote education, employment, family, and community participation as central outcomes, consistent with evidence of persistent reintegration and employment barriers across LMICs [30,32,33,48–50].

## Dynamic interactions across framework levels

Rehabilitation outcomes emerge from dynamic interactions across levels: economic deterioration may accelerate workforce migration, reducing service availability and continuity and increasing complication risk [33,51]; weak implementation of disability legislation may compound environmental and transportation barriers limiting participation despite successful clinical rehabilitation [33,52,53]. These feedback loops illustrate how fragility generates cascading effects throughout the rehabilitation continuum, consistent with challenges documented elsewhere [35,36], reinforcing the need for coordinated, multi-level action.

## Discussion

This paper addresses a gap in rehabilitation systems research: existing frameworks (e.g. WHO Rehabilitation 2030) provide limited guidance where governance, financing, and workforce capacity are severely constrained [34,54]. The Lebanese context shows how fragmentation, financial hardship, and workforce depletion disrupt continuity and outcomes for people with SCI, echoing evidence from other LMICs and fragile settings [33,38].

The proposed framework extends existing approaches by explicitly treating health system fragility as a cross-cutting determinant – rather than a background condition – shaping governance, service delivery, and functioning. Relative to WHO Rehabilitation 2030 and the WHO Health System Building Blocks, which largely assume stable infrastructure and treat system components independently, the framework foregrounds their dynamic interaction under fragility; relative to Community-Based Rehabilitation, often implemented in parallel to formal services, it integrates community participation within the governance and service-delivery architecture. A full comparison against these and existing SCI-specific models is in Supplementary Table S5.

A key implication is the need for adaptive, coordinated strategies: telehealth and telerehabilitation may help mitigate workforce shortages and service fragmentation by supporting continuity and specialist consultation in resource-constrained settings [39,40,55], though these approaches cannot substitute for comprehensive rehabilitation services.

Recommended actions can be organized by implementation horizon: short term (0–2 years) priorities include multisectoral coordination and digital-health pilots; medium term (2–5 years) priorities include sustainable financing and workforce retention; long term (5+ years) priorities include full legislative implementation and UHC integration – reflecting that structural reforms require longer timeframes than service-level innovations.

Although developed in Lebanon, the framework may apply to other LMICs and protracted-crisis settings facing similar governance, financing, and workforce challenges [29], serving as a conceptual tool rather than a prescriptive model.

## Conclusion

SCI rehabilitation in Lebanon illustrates how health system fragility affects rehabilitation access, continuity, and participation. The proposed ICF-informed, multi-level framework integrates governance, service delivery, and community participation domains while accounting for contextual instability. Although developed for Lebanon, it may inform rehabilitation planning in other fragile, resource-constrained settings. As an expert-informed conceptual contribution rather than a fully validated model, it requires future empirical and stakeholder validation to confirm its applicability and scalability across diverse fragile and crisis-affected settings.

## Supplementary Material

Supplementary_Materials_de.docx

## Data Availability

No primary data were generated or analyzed in this study. The paper is based on a narrative literature synthesis, policy analysis, and conceptual framework development.

## Preprints

This manuscript has not been posted to a preprint server.
