# Supplementary material for: Reframing spinal cord injury rehabilitation in fragile contexts: a multi-level systems framework for Lebanon
Source: Glob Health Action. 2026 Aug 17;19(1):2711533. doi: 10.1080/16549716.2026.2711533 (PMC13482921; doi:10.1080/16549716.2026.2711533)
Supplement: Supplementary file 1 [file ZGHA_A_2711533_SM0692.docx]

**Supplementary Material**

Reframing Spinal Cord Injury Rehabilitation in Fragile Contexts: A Multi-Level Systems Framework for Lebanon (Current Debate)

## ***Supplementary Material S1: PubMed Search Strategy***

*(("Spinal Cord Injuries"[Mesh] OR "spinal cord injury"[Title/Abstract] OR "spinal cord injuries"[Title/Abstract] OR SCI[Title/Abstract])) AND (rehabilitation[Mesh] OR rehabilitation[Title/Abstract] OR "rehabilitation services"[Title/Abstract] OR "community-based rehabilitation"[Title/Abstract]) AND (governance[Title/Abstract] OR financing[Title/Abstract] OR workforce[Title/Abstract] OR "health system*"[Title/Abstract] OR "service delivery"[Title/Abstract] OR "continuity of care"[Title/Abstract] OR participation[Title/Abstract] OR disability[Title/Abstract] OR inclusion[Title/Abstract]) AND (LMIC*[Title/Abstract] OR "low- and middle-income countr*"[Title/Abstract] OR "developing countr*"[Title/Abstract] OR "fragile health system*"[Title/Abstract] OR "fragile state*"[Title/Abstract] OR "conflict-affected"[Title/Abstract] OR "crisis-affected"[Title/Abstract] OR humanitarian[Title/Abstract])*

## ***Supplementary Table S2: Evidence Sources Included in the Literature Synthesis (n = 35)***

| **PMID** | **Citation** |
| --- | --- |
| 41712819 | Salah S, Ben Saad H, Moncer R, Gaddour M, Jemni S, Ben Salah Frih Z. Management of Spinal Cord Injury in Tunisia: 2024 landscape and challenges. Tunis Med. 2025;103(4). doi:10.62438/tunismed.v103i4.5496. |
| 41528942 | Gao F, Liu W, Deng C, Wang Z, Wang B, Deng H, Liu Y, Sun L. The Global Macroeconomic Burden of Spinal Cord Injury: A Dynamic Modeling Study from 2020 to 2050. Neuroepidemiology. 2026. doi:10.1159/000550343. |
| 36803149 | Zimmerman A, Minnig MC, Meela J, Tupetz A, Bettger JP, Vissoci JRN, Staton C. A systematic review and cross-sectional survey of rehabilitation resources for injury patients in the Kilimanjaro Region of Tanzania. Disabil Rehabil. 2024. doi:10.1080/09638288.2023.2179674. |
| 29582900 | Renaud R, Locke HN, Hariharan R, Chamberlain MA, O'Connor RJ. Developing a spinal cord injury rehabilitation service in Madagascar. J Rehabil Med. 2018;50(5):402-5. doi:10.2340/16501977-2323. |
| 30633693 | Dorjbal D, Prodinger B, Zanini C, Avirmed B, Stucki G, Rubinelli S. Living with spinal cord injury in Mongolia: A qualitative study on perceived environmental barriers. J Spinal Cord Med. 2020. doi:10.1080/10790268.2019.1565707. |
| 30774289 | Alve YA, Bontje P. Factors Influencing Participation in Daily Activities by Persons With Spinal Cord Injury: Lessons Learned From an International Scoping Review. Top Spinal Cord Inj Rehabil. 2019;25(1):41-61. doi:10.1310/sci2501-41. |
| 29616278 | Furlan AD, Irvin E, Munhall C, Giraldo-Prieto M, Fullerton L, McMaster R, et al. Rehabilitation service models for people with physical and/or mental disability living in low- and middle-income countries: A systematic review. J Rehabil Med. 2018. doi:10.2340/16501977-2325. |
| 11571955 | Basso A, Previgliano I, Duarte JM, Ferrari N. Advances in management of neurosurgical trauma in different continents. World J Surg. 2001. doi:10.1007/s00268-001-0079-3. |
| 41154231 | Kim M, Jeong W, Jang S, Park JH, Bae Y, Lee SW. Spinal Cord Injury Epidemiology and Causes: A Worldwide Analysis with 2050 Projections. Healthcare (Basel). 2025;13(20):2552. doi:10.3390/healthcare13202552. |
| 35892134 | Abu Mostafa MK, Plastow NA, Savin-Baden M. Participatory Methods to Develop Health Education for PW-SCI: Perspectives on Occupational Justice. Can J Occup Ther. 2023. doi:10.1177/00084174221116250. |
| 42165224 | Abumostafa MK, Plastow NA, Savin-Baden M. Participation Education Intervention for People With SCI From LMIC: A Pilot Randomized Clinical Trial. Occup Ther Int. 2026. doi:10.1155/oti/8774897. |
| 35523952 | Uddin T, Shakoor MA, Rathore FA, Sakel M. Ethical issues and dilemmas in spinal cord injury rehabilitation in the developing world: a mixed-method study. Spinal Cord. 2022. doi:10.1038/s41393-022-00808-8. |
| 42211036 | Singh I. Dignified Moving and Handling in Spinal Cord Injury Rehabilitation: A Global Call for Practice Transformation. Top Spinal Cord Inj Rehabil. 2026. doi:10.46292/sci25-00079. |
| 36278119 | Dhakal R, Baniya M, Solomon RM, Rana C, Ghimire P, Hariharan R, et al. TEleRehabilitation Nepal (TERN) for People With Spinal Cord Injury and Acquired Brain Injury: A Feasibility Study. Rehabil Process Outcome. 2022;11. doi:10.1177/11795727221126070. |
| 33304205 | Yao DPG, Inoue K, Sy MP, Bontje P, Suyama N, Yatsu C, Perez DA, Ito Y. Experience of Filipinos with Spinal Cord Injury in the Use of Assistive Technology: An Occupational Justice Perspective. Occup Ther Int. 2020. doi:10.1155/2020/6696296. |
| 26696466 | Doyle ST, Perrin PB, Nicholls E, Olivera SL, Quintero LM, Otálvaro NY, Arango-Lasprilla JC. Pediatric SCI/D caregiver mental health and family dynamics in Colombia, South America. Disabil Rehabil. 2016. doi:10.3109/09638288.2015.1046568. |
| 31632728 | Tyagi N, Amar Goel S, Alexander M. Improving quality of life after spinal cord injury in India with telehealth. Spinal Cord Ser Cases. 2019. doi:10.1038/s41394-019-0212-x. |
| 39595510 | O'Connell C, Armstrong J, De la Cerna-Luna R, Ganvir S, Arnillas Brigneti P. Perspective of the World Rehabilitation Alliance: Global Strategies to Strengthen Spinal Cord Injury Rehabilitation Services in Health Systems. Healthcare (Basel). 2024;12(22). doi:10.3390/healthcare12222313. |
| 31790290 | Dorjbal D, Zanini C, Tsegmid N, Stucki G, Rubinelli S. Toward an optimization of rehabilitation services for persons with spinal cord injury in Mongolia: the perspective of medical doctors. Disabil Rehabil. 2021;43(15):2200-12. doi:10.1080/09638288.2019.1696415. |
| 31023347 | Herbert RD, Harvey LA, Hossain MS, Islam MS, Li Q, Billot L; CIVIC Trial Collaboration. Community-based interventions to prevent serious complications following spinal cord injury in Bangladesh: the CIVIC trial statistical analysis plan. Trials. 2019. doi:10.1186/s13063-019-3181-2. |
| 26238316 | Derakhshanrad N, Vosoughi F, Yekaninejad MS, Moshayedi P, Saberi H. Functional impact of multidisciplinary outpatient program on patients with chronic complete spinal cord injury. Spinal Cord. 2015. doi:10.1038/sc.2015.136. |
| 28271725 | Ferdiana A, Post MWM, King N, Bültmann U, van der Klink JJL. Meaning and components of quality of life among individuals with spinal cord injury in Yogyakarta Province, Indonesia. Disabil Rehabil. 2018. doi:10.1080/09638288.2017.1294204. |
| 39268851 | Kaka B, Sulaiman SK, Mohammad AH, Bello B, Abba MA, Bello UM, Salihu D, Chutiyami M, Zandam H, Fatoye F. Perception of community reintegration among individuals with spinal cord injury in northwest Nigeria: a qualitative description. Disabil Rehabil. 2025. doi:10.1080/09638288.2024.2402073. |
| 41531887 | Vlok AJ, Tamai K, Alassiri SS, Blattert TR, Campello MA, Dunn RN, et al. SPINE20 recommendations 2025: Sustainable spine care for all. Brain Spine. 2026;6:105886. doi:10.1016/j.bas.2025.105886. |
| 21187218 | Rathore FA, New PW, Iftikhar A. A report on disability and rehabilitation medicine in Pakistan: past, present, and future directions. Arch Phys Med Rehabil. 2011. doi:10.1016/j.apmr.2010.10.004. |
| 18333519 | Rathore MF, Hanif S, Farooq F, Ahmad N, Mansoor SN. Traumatic spinal cord injuries at a tertiary care rehabilitation institute in Pakistan. J Pak Med Assoc. 2008. |
| 32003248 | Moshi H, Sundelin G, Sahlen KG, Sörlin A. Quality of life of persons with traumatic spinal cord injury in rural Kilimanjaro, Tanzania: a community survey. Disabil Rehabil. 2021. doi:10.1080/09638288.2020.1718780. |
| 40637549 | Bhattarai M, Karki S, Khadka A, Baniya M, Dhakal R. A qualitative study uncovering rehabilitation challenges: Perspectives of individuals with spinal cord injury. J Spinal Cord Med. 2026. doi:10.1080/10790268.2025.2526252. |
| 32372030 | Pongpipatpaiboon K, Selb M, Kovindha A, Prodinger B. Toward a framework for developing an ICF-based documentation system in spinal cord injury-specific rehabilitation based on routine clinical practice: a case study approach. Spinal Cord Ser Cases. 2020. doi:10.1038/s41394-020-0283-8. |
| 31980830 | Ehsanian R, Creasey G, Elliott CS, Abu-Eid CA, Ali A, Prutton M, Singh H. Implantation of Sacral Nerve Stimulator Without Rhizotomy for Neurogenic Bladder in Patient With Spinal Cord Injury: 2-Dimensional Operative Video. Oper Neurosurg. 2020. doi:10.1093/ons/opz429. |
| 39419920 | Ullah MM, Fossey E, Stuckey R. Meaning of Work Participation After Spinal Cord Injury in Bangladesh: A Qualitative Study in a Low- and Middle-Income Country Context. J Occup Rehabil. 2025. doi:10.1007/s10926-024-10244-5. |
| 40853689 | Tyagi N, Rathore FA, Koly K, Mashola MK, Dhakal R, Evans RW, Albertus Y. The use of telehealth in the management of people with spinal cord injury in Low-Middle-Income Countries during COVID-19 pandemic. Work. 2026;83(2):432-9. doi:10.1177/10519815251368308. |
| 40678088 | Mullerpatan R, Ryain A, Padia N, Salgotra KR. Patient-reported challenges in spinal cord injury rehabilitation and integration within the community of a lower middle-income country: opportunities for changing lives. Brain Spine. 2025. doi:10.1016/j.bas.2025.104299. |
| 26694314 | Löfvenmark I, Norrbrink C, Nilsson Wikmar L, Löfgren M. 'The moment I leave my home - there will be massive challenges': experiences of living with a spinal cord injury in Botswana. Disabil Rehabil. 2016. doi:10.3109/09638288.2015.1106596. |
| 22145085 | Shamim MS, Ali SF, Enam SA. Non-operative management is superior to surgical stabilization in spine injury patients with complete neurological deficits: A perspective study from a developing world country, Pakistan. Surg Neurol Int. 2011. doi:10.4103/2152-7806.90027. |

## ***Supplementary Table S3: Policy, Contextual, and Conceptual Sources Reviewed (n = 10)***

| **Document** | **Organization** | **Contribution to Framework** |
| --- | --- | --- |
| Law 220/2000 on the Rights of Persons with Disabilities | Government of Lebanon | Informed disability rights, accessibility, and social inclusion and participation domains. |
| Rehabilitation in Lebanon (2023) | Handicap International | Provided evidence on rehabilitation service organization, accessibility barriers, and system fragmentation. |
| Physical and Functional Rehabilitation in Lebanon (2022) | Handicap International | Informed rehabilitation service delivery challenges and workforce considerations. |
| Forgotten and Invisible: The Impact of Lebanon's Crises on Persons with Disabilities (2023) | Handicap International | Contributed evidence on disability inclusion, social participation barriers, and the effects of health system fragility. |
| Rehabilitation 2030: A Call for Action | World Health Organization (WHO) | Informed rehabilitation system strengthening, governance, financing, and workforce development domains. |
| International Classification of Functioning, Disability and Health (ICF) | World Health Organization (WHO) | Served as the conceptual framework guiding the organization of functioning, environmental factors, and participation outcomes. |
| Universal Health Coverage and Rehabilitation-related WHO guidance documents | World Health Organization (WHO) | Informed integration of rehabilitation within broader health systems and financing mechanisms. |
| Healthcare Delivery in Lebanon: A Critical Scoping Review of Strengths, Weaknesses, Opportunities, and Threats | Published health systems review | Provided contextual evidence regarding governance challenges, privatization, workforce migration, and health system fragility in Lebanon. |
| Building Hope: Early Rehabilitation Response of the International Committee of the Red Cross Post Beirut Port Blast | International Committee of the Red Cross (ICRC) | Informed understanding of rehabilitation service provision during humanitarian crises and emergency contexts. |
| National reports and publicly available documents from the Lebanese Ministry of Public Health (MOPH) | Ministry of Public Health, Lebanon | Provided contextual information regarding healthcare organization, financing, and rehabilitation service delivery in Lebanon. |

## ***Supplementary Table S4: Analytical Mapping of Evidence Sources and Thematic Findings***

*Analytical mapping of evidence sources and thematic findings informing the development of the proposed SCI rehabilitation framework for fragile health systems.*

| **Analytical Input** | **Key Contextual and Empirical Insights** | **Framework Domain Derived** |
| --- | --- | --- |
| Contextual policy analysis (governance, financing, and legal environment) | - Weak enforcement of disability legislation (Law 220/2000) and absence of operational implementation mechanisms. - Fragmented and highly privatized healthcare system characterized by high out-of-pocket expenditures and donor-dependent rehabilitation services [10, 11, 21]. - Limited national coordination and lack of long-term rehabilitation planning [11, 21]. - Global evidence highlights the need for stronger rehabilitation governance, sustainable financing mechanisms, and integration of SCI rehabilitation within national health systems [9, 31]. | Macro-level structural domain: Addresses governance arrangements, financing mechanisms, legislative implementation, and multisectoral coordination that influence rehabilitation access, equity, and long-term system sustainability. |
| Structured literature synthesis (service organization and workforce capacity) | - Workforce migration ("brain drain") reducing the availability of specialized rehabilitation professionals [12, 13, 14, 15]. - Urban concentration of rehabilitation services with limited access for rural and underserved populations [21]. - Fragmented rehabilitation pathways and weak continuity between acute, post-acute, and community-based services [24, 25]. - Similar challenges have been reported in Tunisia, Tanzania, Mongolia, Madagascar, and Pakistan, where shortages of specialized personnel and rehabilitation infrastructure constrain service delivery [5, 6, 7, 8, 22, 23, 39]. | Meso-level health system domain: Addresses service organization, interdisciplinary workforce capacity, continuity of care, rehabilitation integration, information systems, and innovation strategies within fragile health systems. |
| ICF-informed conceptual integration (functioning, participation, and environmental interaction) | - Preventable secondary complications linked to inadequate follow-up and fragmented post-discharge care [26, 27]. - Environmental inaccessibility, disability stigma, and insufficient psychosocial support limiting participation and social reintegration [17]. - Participation restrictions, reduced autonomy, and limited self-management capacity among individuals with SCI [17, 28]. - Evidence from Mongolia, Botswana, Bangladesh, Nigeria, Indonesia, and international reviews consistently identifies environmental barriers, reduced community participation, work exclusion, and limited social reintegration as major determinants of long-term outcomes after SCI [24, 50, 51, 52, 53, 54, 55]. | Micro-level functioning and participation domain: Focuses on individual functioning, self-management, psychosocial support, assistive technology access, environmental accessibility, and community participation outcomes. |
| Cross-cutting synthesis across all evidence streams | - Chronic political and economic instability undermining financing, workforce retention, institutional effectiveness, and continuity of rehabilitation services across all system levels [35]. - Structural fragility affecting governance effectiveness, rehabilitation continuity, workforce retention, and the sustainability of rehabilitation interventions [40]. - Telehealth and telerehabilitation initiatives from Nepal, India, and other LMICs demonstrate how innovation may partially mitigate fragility-related disruptions in workforce availability and service continuity [41, 42, 43]. | Cross-cutting determinant: Health system fragility. Conceptualizes fragility as an active structural condition influencing governance, service delivery, rehabilitation continuity, and long-term functioning outcomes across the framework. |

## ***Supplementary Table S5: Comparison of the Proposed Framework with Existing Rehabilitation and Health Systems Frameworks***

| **Framework** | **Primary Focus** | **Treatment of System Stability / Fragility** | **Relationship to Proposed Framework** |
| --- | --- | --- | --- |
| WHO Rehabilitation 2030 | Expanding rehabilitation workforce, service coverage, and integration within UHC | Assumes relatively stable governance and financing systems | Extends this agenda by explicitly foregrounding fragility as a cross-cutting determinant rather than a background condition |
| WHO Health System Building Blocks | Organizes six system components (governance, financing, workforce, information, service delivery, technologies) | Treats components largely independently, with limited attention to dynamic interaction under stress | Emphasizes dynamic, bidirectional interactions among components under fragility, illustrating cascading macro-to-micro effects |
| Community-Based Rehabilitation (CBR) | Grassroots, community-level inclusion and participation | Typically implemented in parallel to formal health systems | Integrates community participation (Pillar 3) within governance and health-system-organization architecture (Pillars 1-2), promoting alignment rather than parallel implementation |
| Existing SCI-specific rehabilitation models | Clinical and service-delivery pathways for SCI rehabilitation, largely developed in high-resource settings | Generally assume stable infrastructure, financing, and workforce availability | Explicitly grounded in a fragile, crisis-affected health system context, adding governance and financing dimensions largely absent from most SCI-specific models |

## ***Supplementary Material S6: Framework Development Process (Methodological Audit Trail)***

| **Component** | **Description** |
| --- | --- |
| Literature database | PubMed |
| Search period | January–March 2026 |
| Search strategy | Full search string provided in Appendix A2 |
| Inclusion criteria | Studies addressing SCI rehabilitation, rehabilitation systems, disability inclusion, health systems, LMICs, and fragile/crisis-affected settings |
| Records reviewed | 35 studies |
| Policy documents reviewed | 10 documents: Law 220/2000, Ministry of Public Health reports, WHO publications, Handicap International reports, and other relevant rehabilitation policy documents (Appendix A4) |
| Thematic domains identified | Governance, financing, workforce capacity, service delivery, participation, disability inclusion, and continuity of care |
| Expert panel | Six authors with expertise in physiotherapy, rehabilitation, public health, disability research, and health systems |
| Consensus process | Three iterative rounds of discussion until agreement on framework domains and relationships |
| Framework structure | Evidence synthesized into macro-level governance and financing, meso-level health system organization and service delivery, and micro-level functioning and participation domains |
